# Supplementary material for: Heat Shock Protein Genes Affect the Rapid Cold Hardening Ability of Two Invasive Tephritids
Source: Insects. 2024 Jan 29;15(2):90. doi: 10.3390/insects15020090 (PMC10889258; doi:10.3390/insects15020090)
Supplement: Supplementary file 1 [file insects-15-00090-s001.zip › supplymentary files.pdf]

Supplementary files

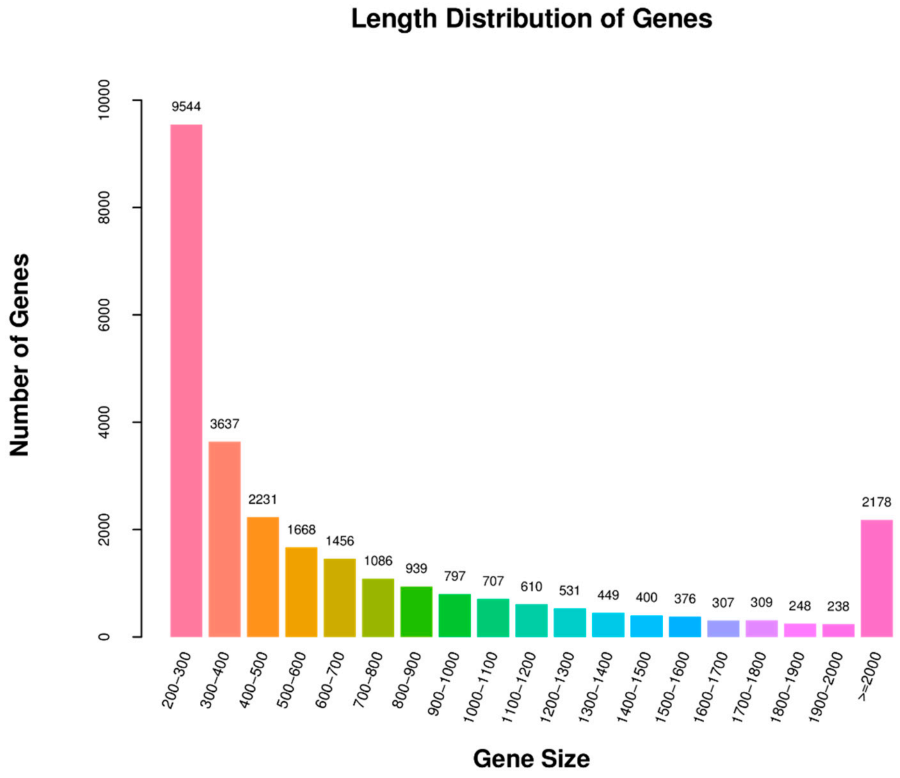

Figure S1 Unigene distribution in transcriptome data.

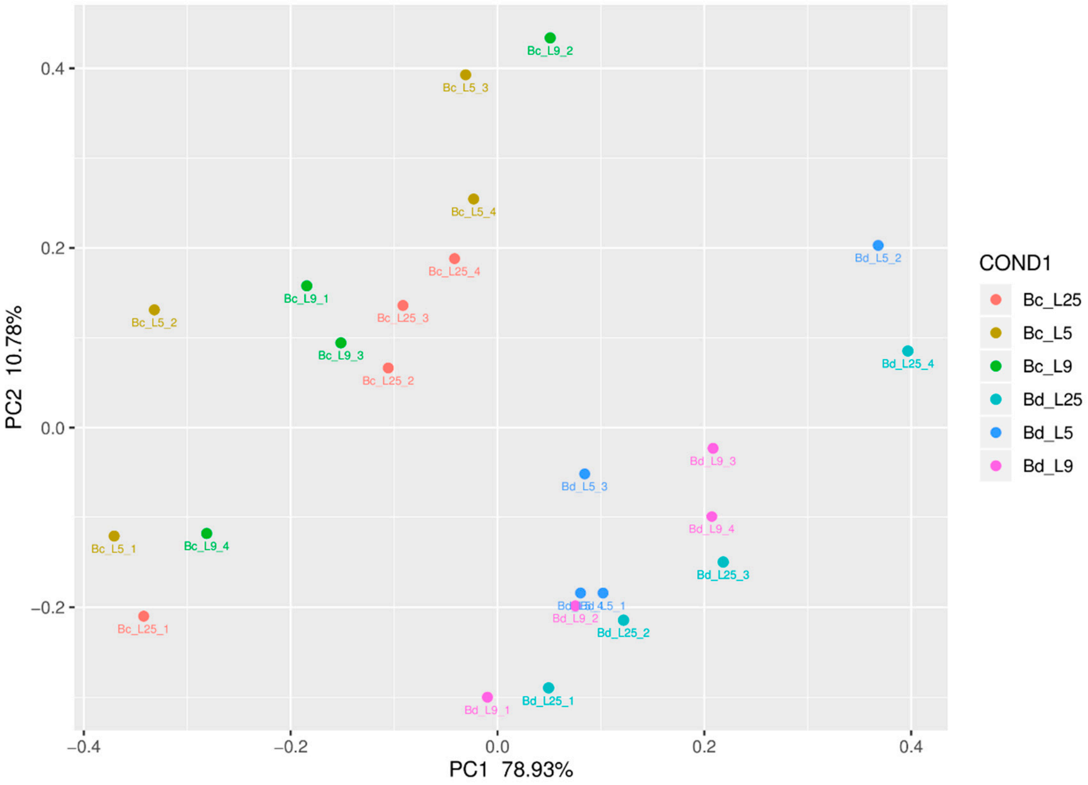

Figure S2 PCA analysis of the samples.

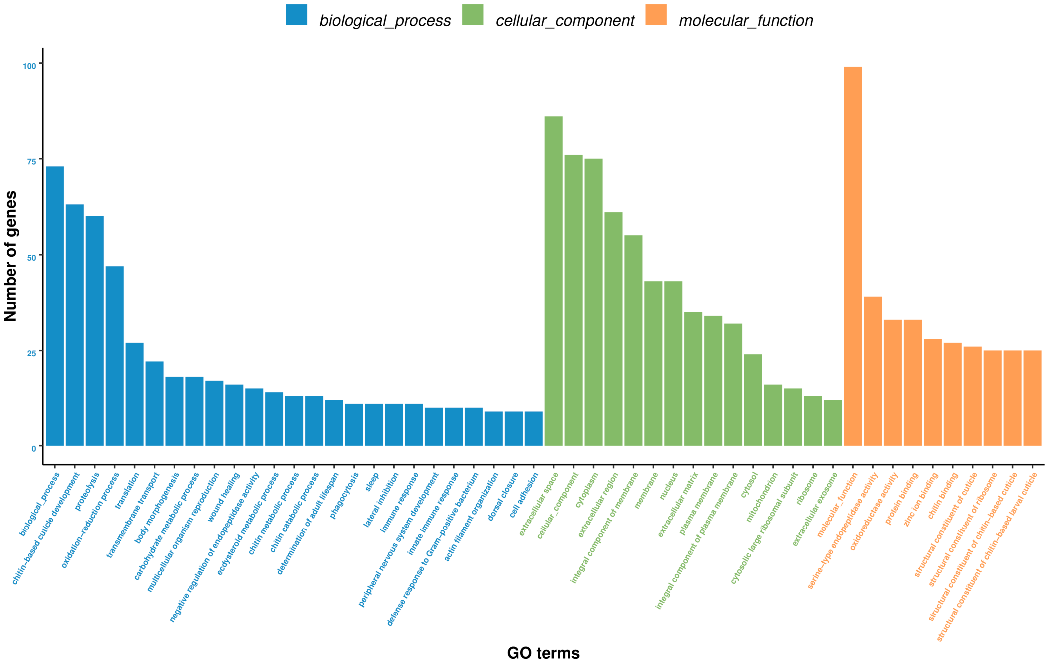

Figure S3 Classification of GO pathways under 5℃ hardening treatments (versus 25℃ control) of *B. dorsalis*.

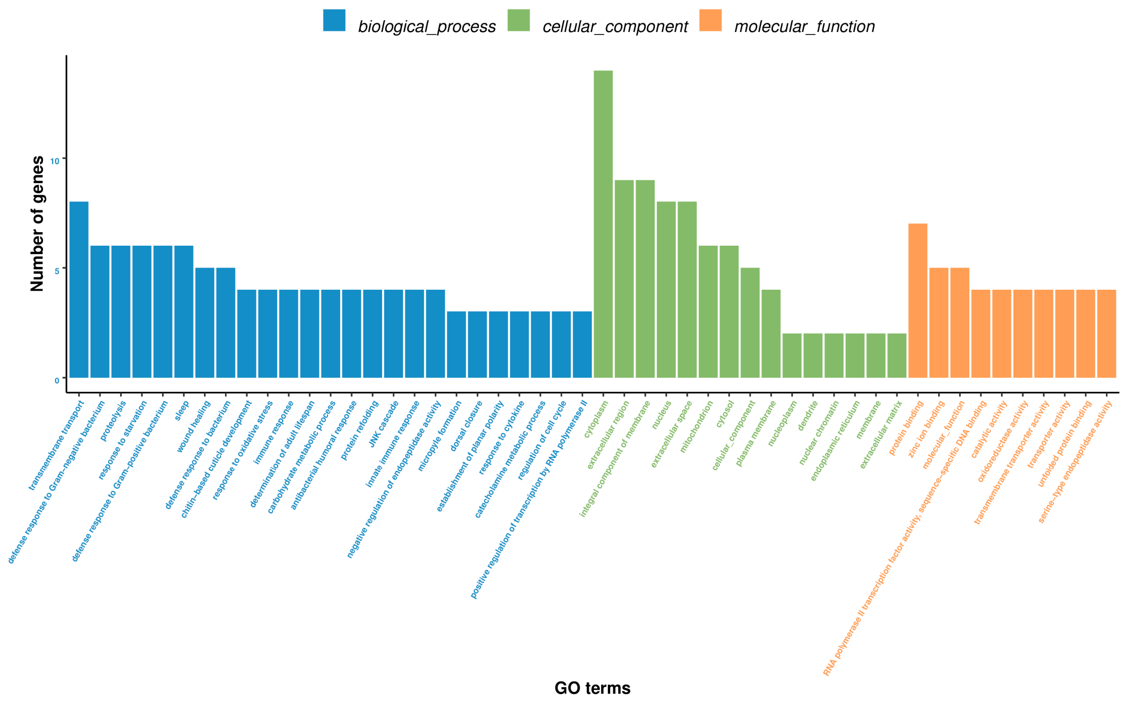

Figure S4 Classification of GO pathways under 9℃ hardening treatments (versus 25℃ control) of *B. dorsalis*.

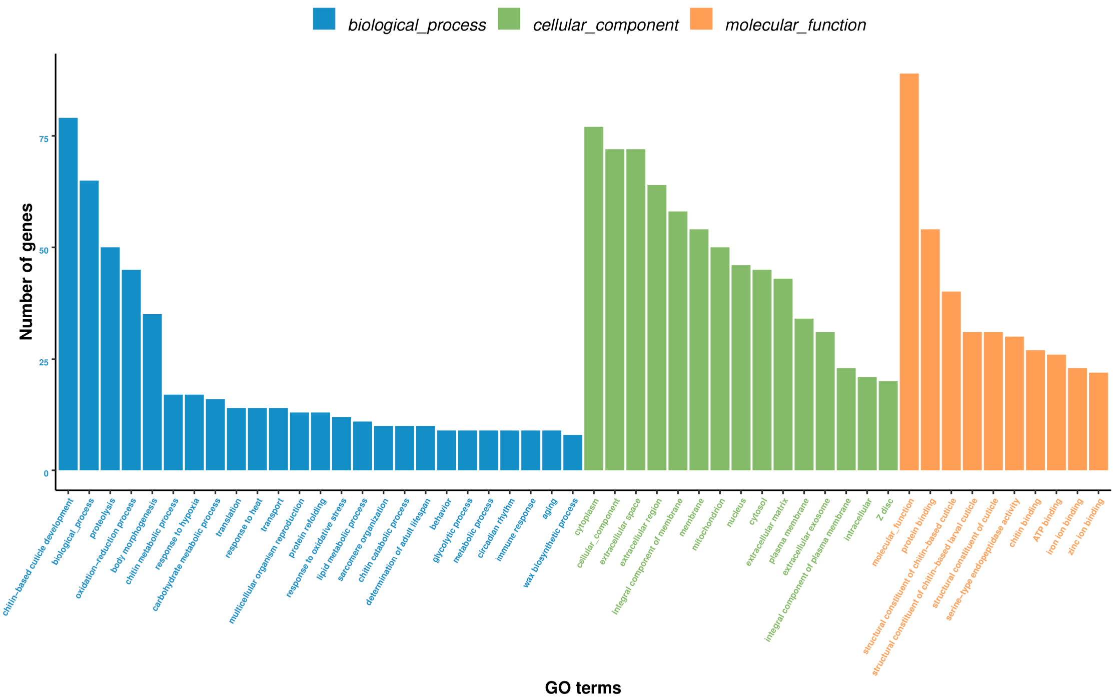

Figure S5 Classification of GO pathways under 5℃ hardening treatments (versus 25℃ control) of *B. correcta.*

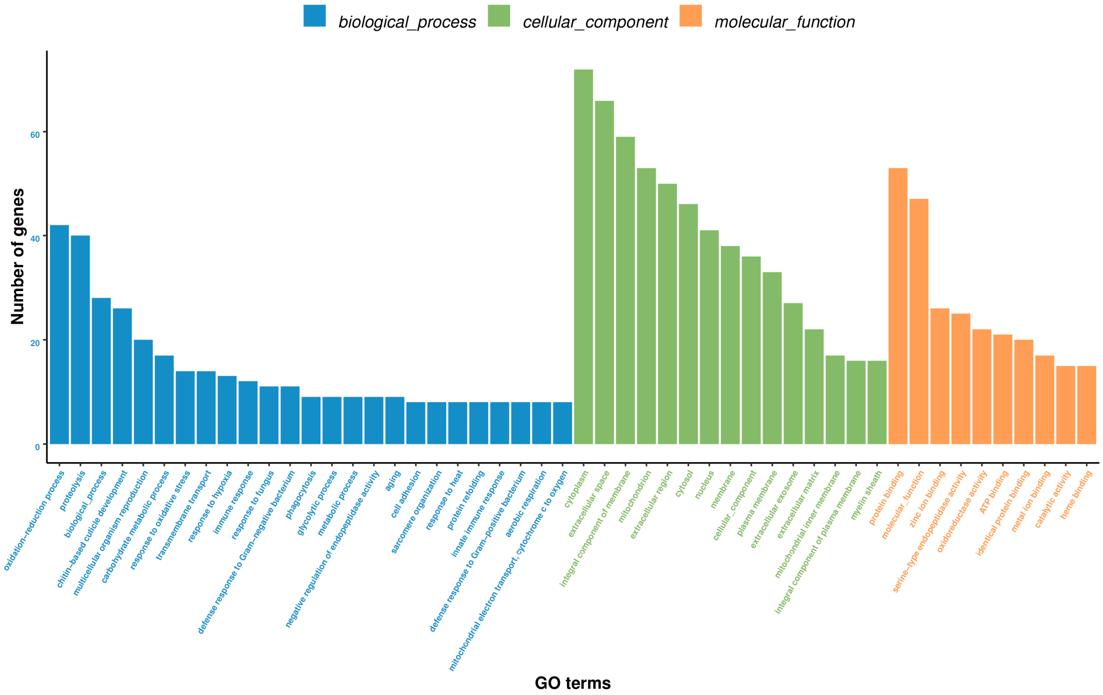

Figure S6 Classification of GO pathways under 9℃ hardening treatments (versus 25℃ control) of *B. correcta.*

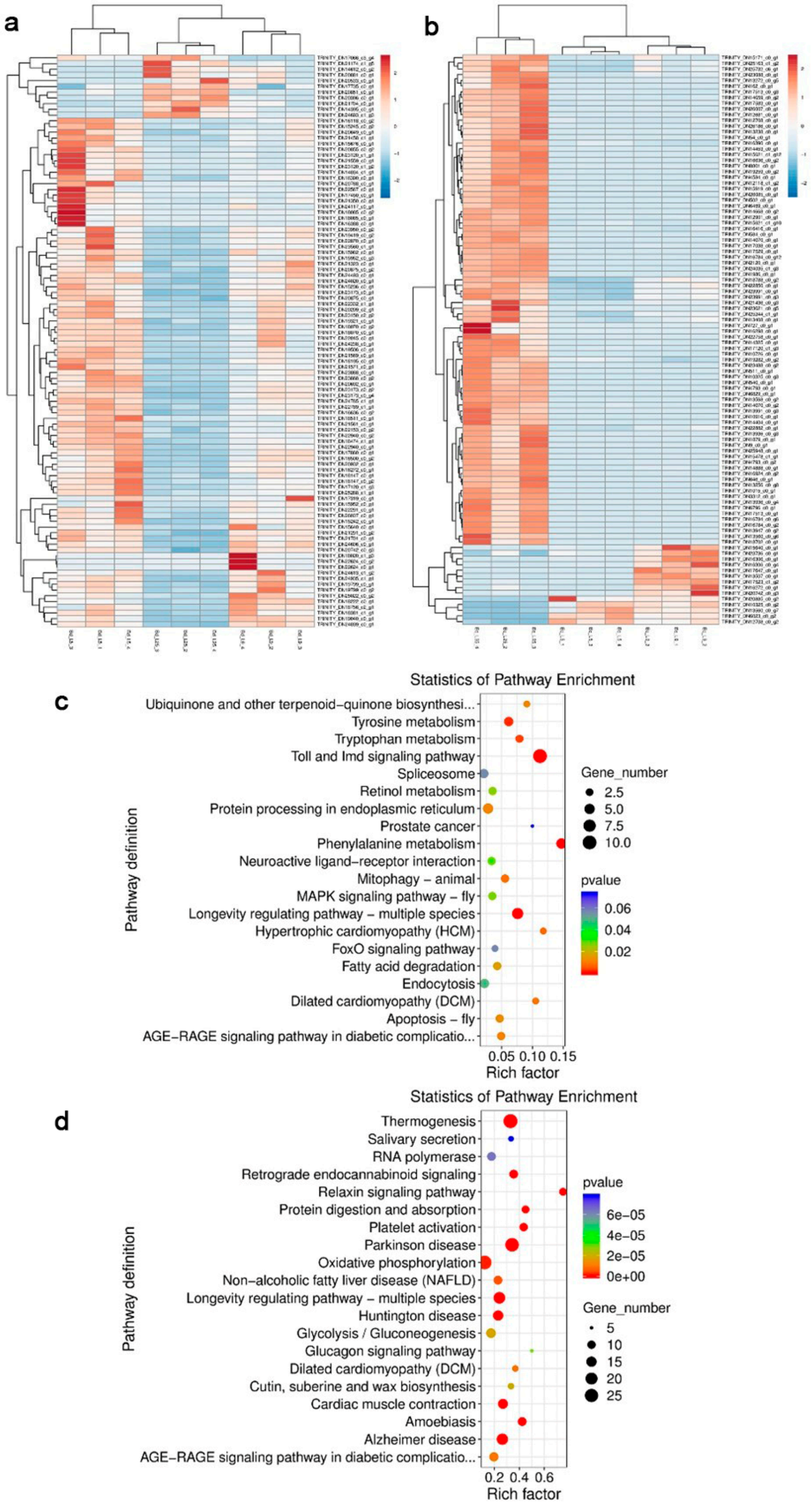

Figure S7 (a) The heatmap of DEGs in *B. dorsalis*. Differential expression is shown as $\log_{10}$ fold changes (LFC) of hardening temperatures versus 25°C using

the value of log10 FPKM for hardening temperatures/FPKM for control temperature. For clarity, LFC values were capped at 1.5 and −1.5. The color bars besides the heatmaps indicate the relative size of the changes. (b) The heatmap of DEGs in *B. correcta*. (c)Classification of KEGG pathways under 9 ℃ hardening treatments (versus 25℃control) of *B. dorsalis*. (d)Classification of KEGG pathways under 5℃ hardening treatments (versus 25℃control) of *B. correcta.*

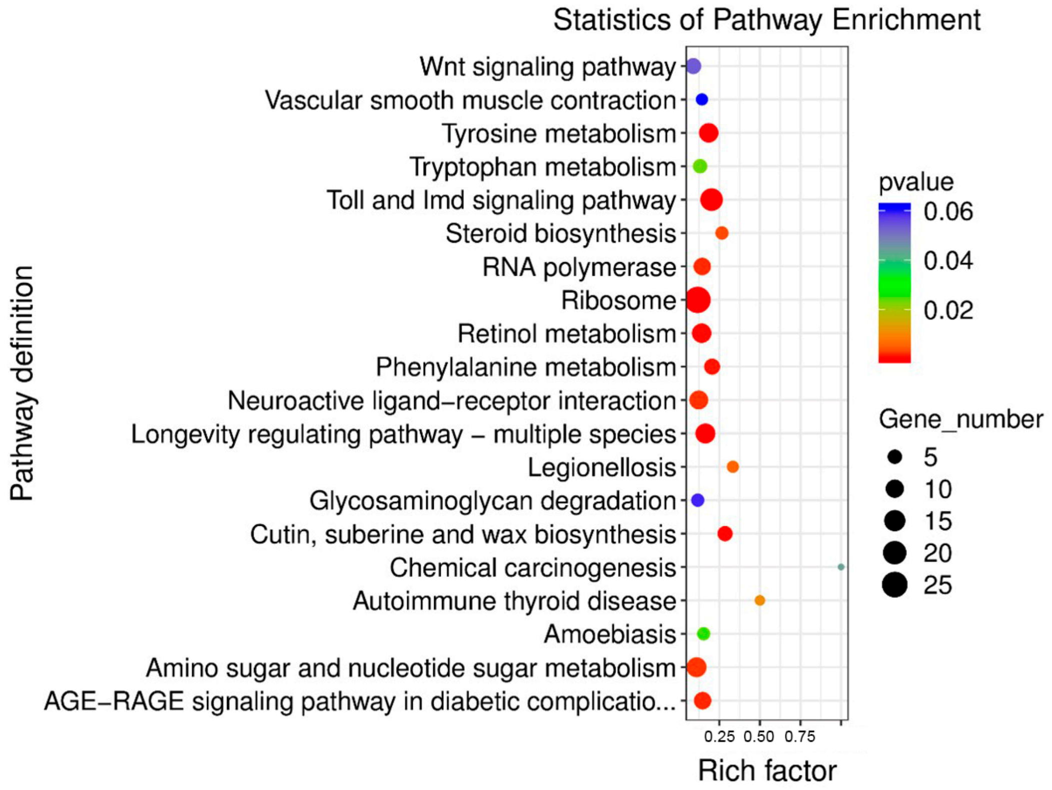

Figure S8 Classification of KEGG pathways under 9℃ hardening treatments (versus 25℃ control) of *B. correcta.*

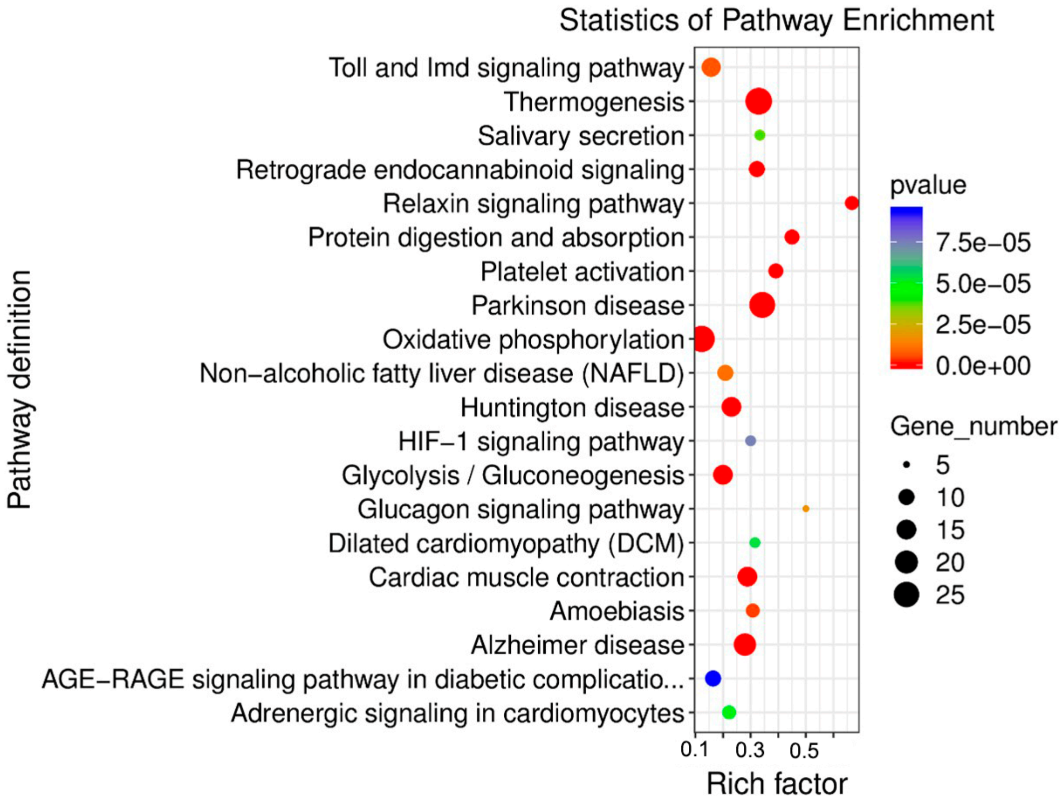

Figure S9 Classification of KEGG pathways under 5℃ hardening treatments (versus 25℃ control) of *B. dorsalis.*

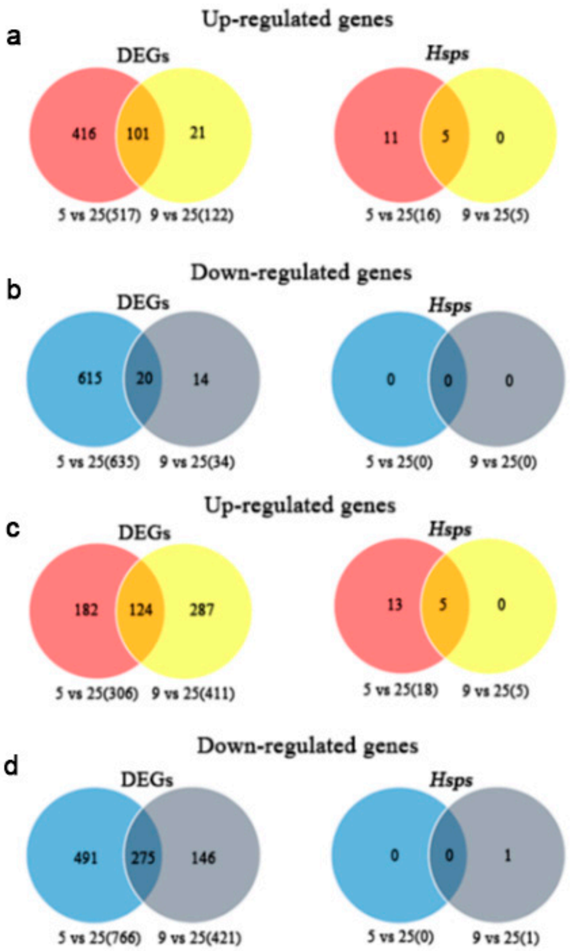

Figure S10 The Venn diagram of different expressed genes in two species. (a) Up-regulated genes and *Hsps* in *B. dorsalis*; (b) Down-regulated genes and *Hsps* in *B. dorsalis*; (c) Up-regulated genes and *Hsps* in *B. correcta*; (b) Down-regulated genes and *Hsps* in *B. correcta*.

Table S1 Primers used in this research. Some genes shared almost same transcript sequences in two *Bactrocera* species, and some of the primers were designed using the same region. The T7 sequence was in red.

Name	Sequence	Description
Hsp23-rt-F	ACACTTTGTTCGCCGCTAT	Primers for *Hsp23* detection in both species
Hsp23-rt-R	GCTTGCCTGCTCCTCAC	
Hsp68-rt-F	AAGCAGCAGACGAAGCGGGAAG	Primers for *Hsp68* detection in both species
Hsp68-rt-R	GCAGTGGTATTGCCATCAAGCCATT	
BdoHsp70-rt-F	ACCAGCATACTTCAATGATT	Primers for *Hsp70* detection in *B. dorsalis*
BdoHsp70-rt-R	TTCGTTAATGATTCGTAGCA	
BcoHsp70-rt-R	GTGACGCTGCGAAGAATCAAGTTG	Primers for *Hsp70* detection in *B. correcta*
BcoHsp70-rt-R	CCAATGCTTCATGTCTGCCTGGAT	
Hsp27-rt-F	ACAGTCAAAGCACCACCACCACCAA	Primers for *Hsp27* detection in both species

Hsp27-rt-R	CTCCTTCGCCTTGCCATCGCTGTTG	
Bdo18S-rt-F	GCGAGAGGTGAAATTCTTGG	Primers for *18s* detection in *B. dorsalis*
Bdo18S-rt-R	CGGGTAAGCGACTGAGAGAG	
Bco18S-rt-F	ACCAGGTCCGAACTTAAGCG	Primers for *18s* detection in *B. correcta*
Bco18S-rt-R	AACCAGACAAATCACTCCACGA	
Hsp23-dsRNA-F-T7	TAATACGACTCACTATAGGATGGCAAACCTACCATTGAT	Primers for *dsHsp23* synthesis with T7 sequence for both species
Hsp23-dsRNA-R-T7	TAATACGACTCACTATAGGTTAAGCGCTTGCCTGCTCCT	
Hsp23-dsRNA-F	ATGGCAAACCTACCATTGAT	Primers for *dsHsp23* synthesis for both species
Hsp23-dsRNA-R	TTAAGCGCTTGCCTGCTCCT	

Hsp68-dsRNA-F-T7	TAATACGACTCACTATAGGGGACGACTTTCCCAAGCAGACATC	Primers for *dsHsp68* synthesis with T7 sequence for both species
Hsp68-dsRNA-R-T7	TAATACGACTCACTATAGGTGTGATTGTGAACCAGCACCTCCT	
Hsp68-dsRNA-F	GGACGACTTTCCCAAGCAGACATC	Primers for *dsHsp68* synthesis for both species
Hsp68-dsRNA-R	TGTGATTGTGAACCAGCACCTCCT	
Hsp70-dsRNA-F-T7	TAATACGACTCACTATAGGTGGTTGCAATTGGAATAGATTTGGG	Primers for *dsHsp70* synthesis with T7 sequence for both species
Hsp70-dsRNA-R-T7	TAATACGACTCACTATAGGATTCAACACTTAACTTGGGCTTTCC	
Hsp70-dsRNA-F	TGGTTGCAATTGGAATAGATTTGGG	Primers for *dsHsp70* synthesis for both species
Hsp70-dsRNA-R	ATTCAACACTTAACTTGGGCTTTCC	

Hsp27-dsRNA-F-T7	TAATACGACTCACTATAGGCCACGTCGTCGCCATTATCCATAC	Primers for *dsHsp27* synthesis with T7 sequence for both species
Hsp27-dsRNA-R-T7	TAATACGACTCACTATAGGCTTCGCCTTGCCATCGCTGTT	
Hsp27-dsRNA-F	CCACGTCGTCGCCATTATCCATAC	Primers for *dsHsp27* synthesis for both species
Hsp27-dsRNA-R	CTTCGCCTTGCCATCGCTGTT	
GFP-dsRNA-T7-F	TAATACGACTCACTATAGGCACAAGTTCAGCGTGTCCG	Primers for *dsGFP* synthesis with T7 sequence for both species
GFP-dsRNA-T7-R	TAATACGACTCACTATAGGGTTCACCTTGATGCCGTTC	
GFP-dsRNA-F	CACAAGTTCAGCGTGTCCG	Primers for *dsGFP* synthesis for both species
GFP-dsRNA-R	GTTCACCTTGATGCCGTTC	

Table S2 Mapped ratios of each sample tested.

Sample	Raw Reads	Raw Bases	Valid Reads	Valid Bases	Valid%	Q20%	Q30%	GC%
Bc_L25_1	46098228	6.91G	45607244	6.39G	98.93	98.67	95.28	43.30
Bc_L25_2	52709828	7.91G	52072508	7.30G	98.79	98.50	94.91	44.20
Bc_L25_3	43808126	6.57G	43423840	6.08G	99.12	98.48	94.84	44.08
Bc_L25_4	49239760	7.39G	48641864	6.81G	98.79	98.42	94.70	44.34
Bc_L5_1	38551256	5.78G	37934266	5.32G	98.40	98.74	95.50	44.00
Bc_L5_2	55220686	8.28G	54479344	7.64G	98.66	98.71	95.40	43.65
Bc_L5_3	55456856	8.32G	54863586	7.69G	98.93	98.70	95.37	44.29
Bc_L5_4	45771100	6.87G	45123222	6.33G	98.58	98.65	95.24	44.62
Bc_L9_1	53388492	8.01G	52698716	7.39G	98.71	98.71	95.40	44.17
Bc_L9_2	47325612	7.10G	46699860	6.55G	98.68	98.70	95.39	44.83
Bc_L9_3	55997438	8.40G	55288452	7.75G	98.73	98.70	95.35	44.09
Bc_L9_4	52717870	7.91G	52043076	7.30G	98.72	98.70	95.38	43.76
Bd_L25_1	49856508	7.48G	49406284	6.93G	99.10	98.72	95.41	44.03
Bd_L25_2	52274336	7.84G	51751396	7.26G	99.00	98.70	95.37	44.12
Bd_L25_3	49167224	7.38G	48722312	6.83G	99.10	98.71	95.38	44.75
Bd_L25_4	53391968	8.01G	52824262	7.41G	98.94	98.75	95.53	45.85
Bd_L5_1	45184386	6.78G	44807064	6.29G	99.16	98.85	95.80	44.14
Bd_L5_2	44363714	6.65G	43986292	6.17G	99.15	98.85	95.83	45.01
Bd_L5_3	41939292	6.29G	41598482	5.84G	99.19	98.82	95.72	44.04
Bd_L5_4	48660106	7.30G	48195724	6.76G	99.05	98.83	95.74	44.19
Bd_L9_1	40442220	6.07G	40014706	5.61G	98.94	98.82	95.70	43.99
Bd_L9_2	54041322	8.11G	53547580	7.51G	99.09	98.72	95.43	44.14
Bd_L9_3	53897152	8.08G	53454302	7.50G	99.18	98.70	95.37	44.46
Bd_L9_4	44756724	6.71G	44306686	6.21G	98.99	98.67	95.30	44.89

Table S3 Statistics for larval unigenes assessment of the two *Bactrocera* species.

Index	All	GC%	Min Length	Median Length	Max Length	Total Assembled Bases	N50
Transcript	81695	40.01	201	402	27315	53922849	953
Gene	27711	40.11	201	425	27315	21115144	1249

Table S4 Summary of gene annotation in the larval transcriptome.

DB	Num	Ratio (%)
All	27711	100.00
GO	11659	42.07
KEGG	9776	35.28
Pfam	10385	37.48
swissprot	8908	32.15
eggNOG	13397	48.35
NR	16771	60.52
