# Supplementary figures and images for: Heat Shock Protein Genes Affect the Rapid Cold Hardening Ability of Two Invasive Tephritids
Source: Insects. 2024 Jan 29;15(2):90. doi: 10.3390/insects15020090 (PMC10889258; doi:10.3390/insects15020090)

## eggNOG functional categories

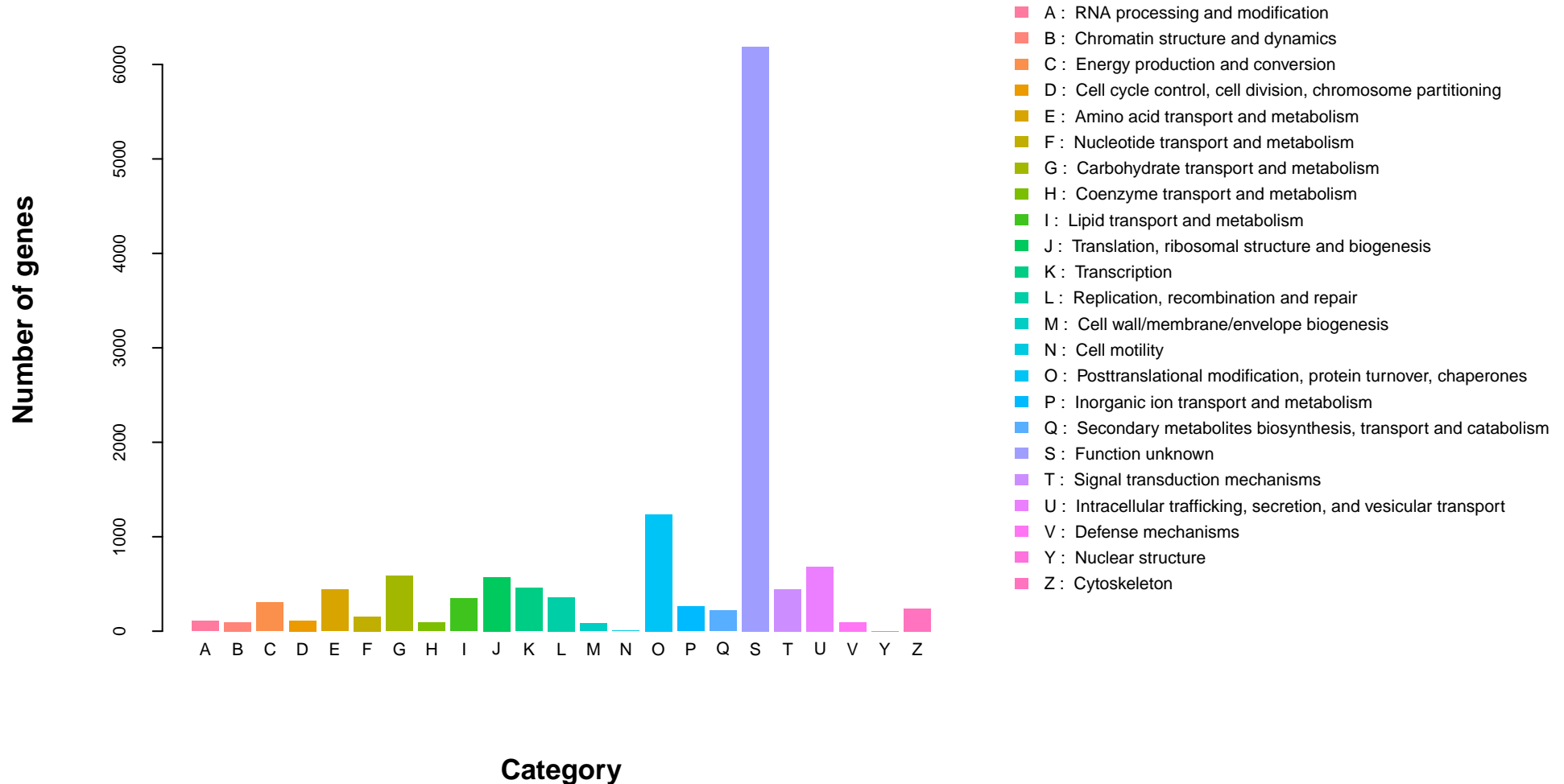

Supplement: Supplementary file 1 [file insects-15-00090-s001.zip › annotation/eggNOG/eggNOG_category.pdf]

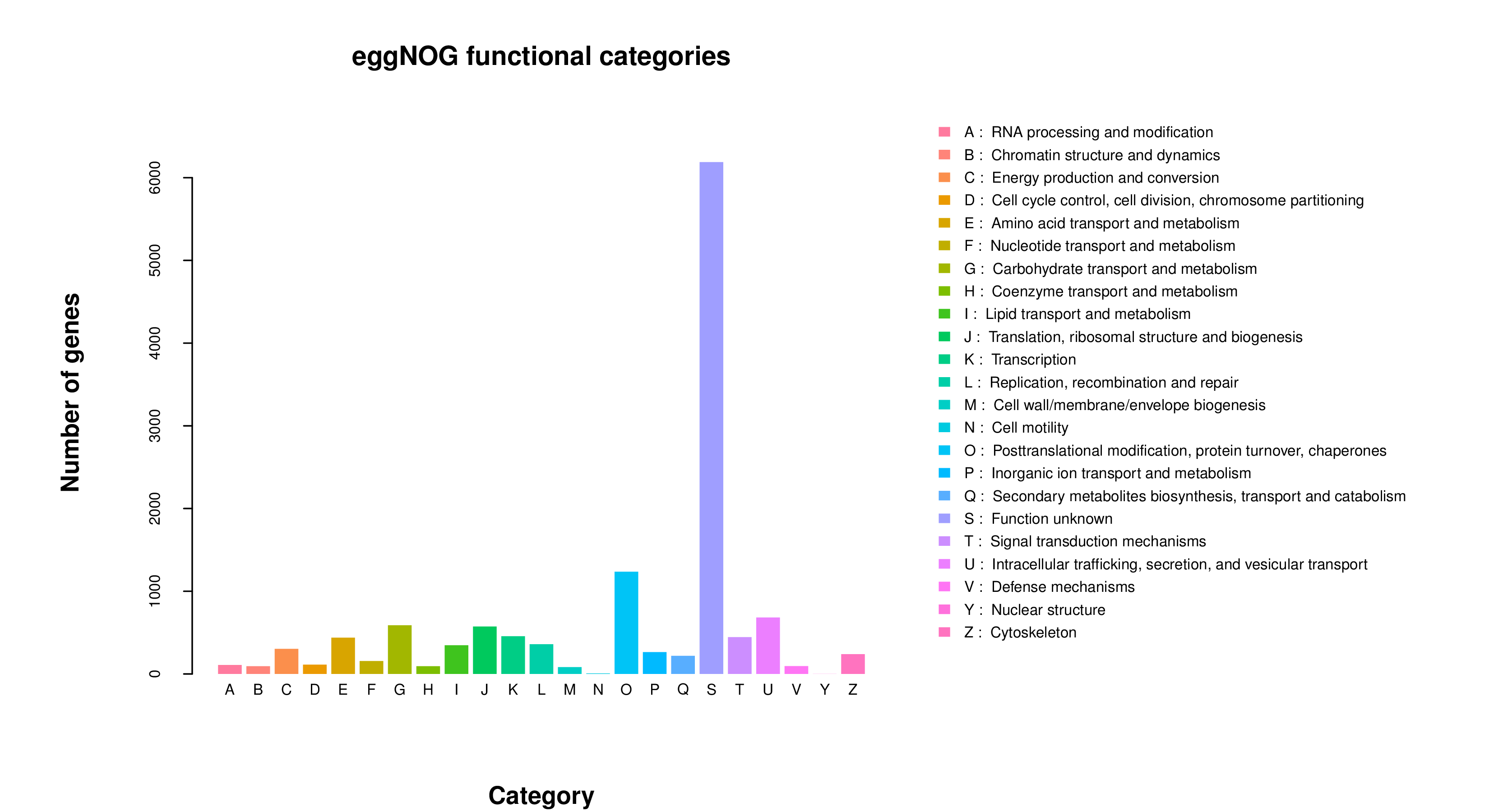

Supplement: Supplementary file 1 [file insects-15-00090-s001.zip › annotation/eggNOG/eggNOG_category.png]

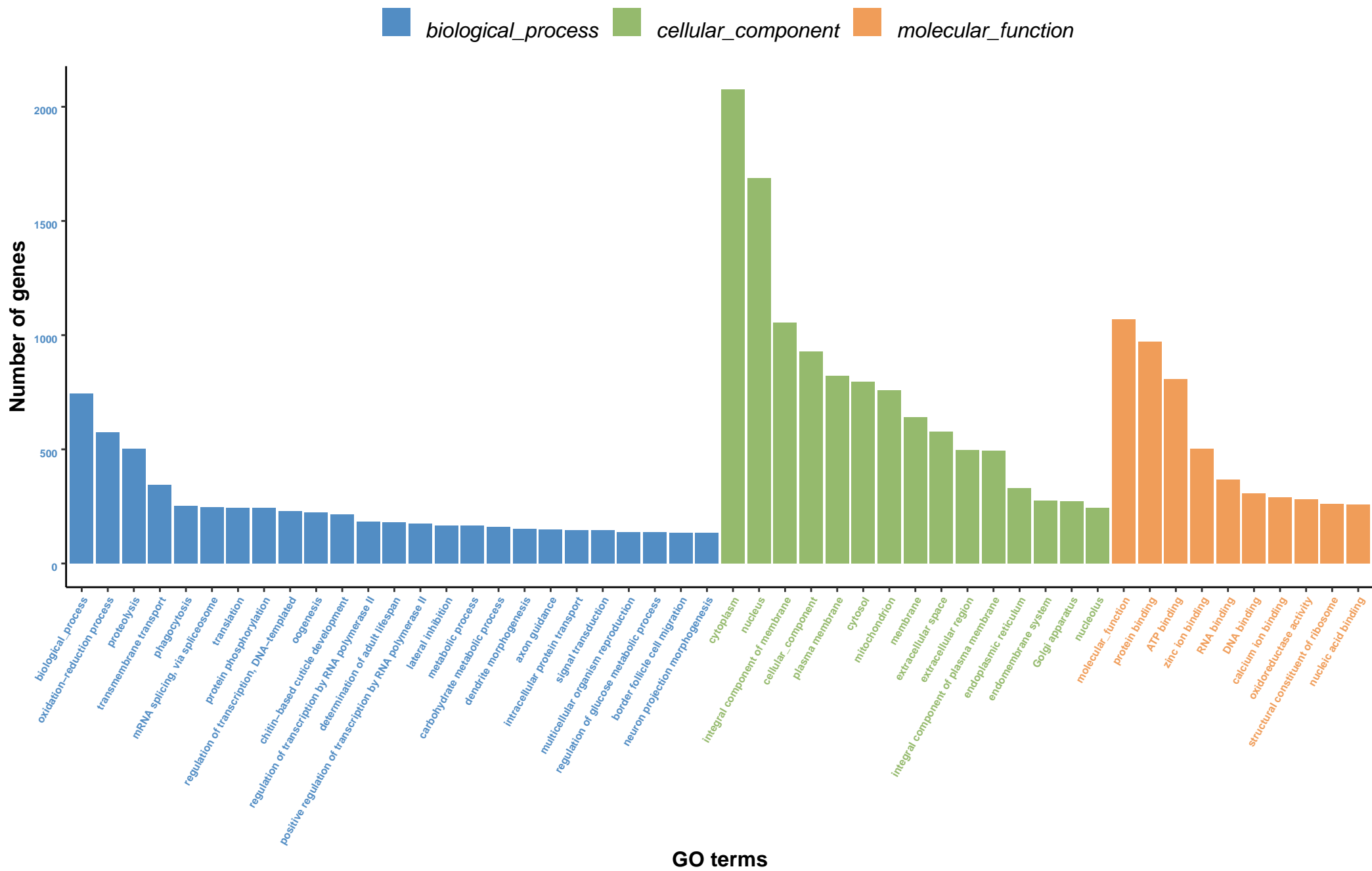

Supplement: Supplementary file 1 [file insects-15-00090-s001.zip › annotation/GO/GO_category.pdf]

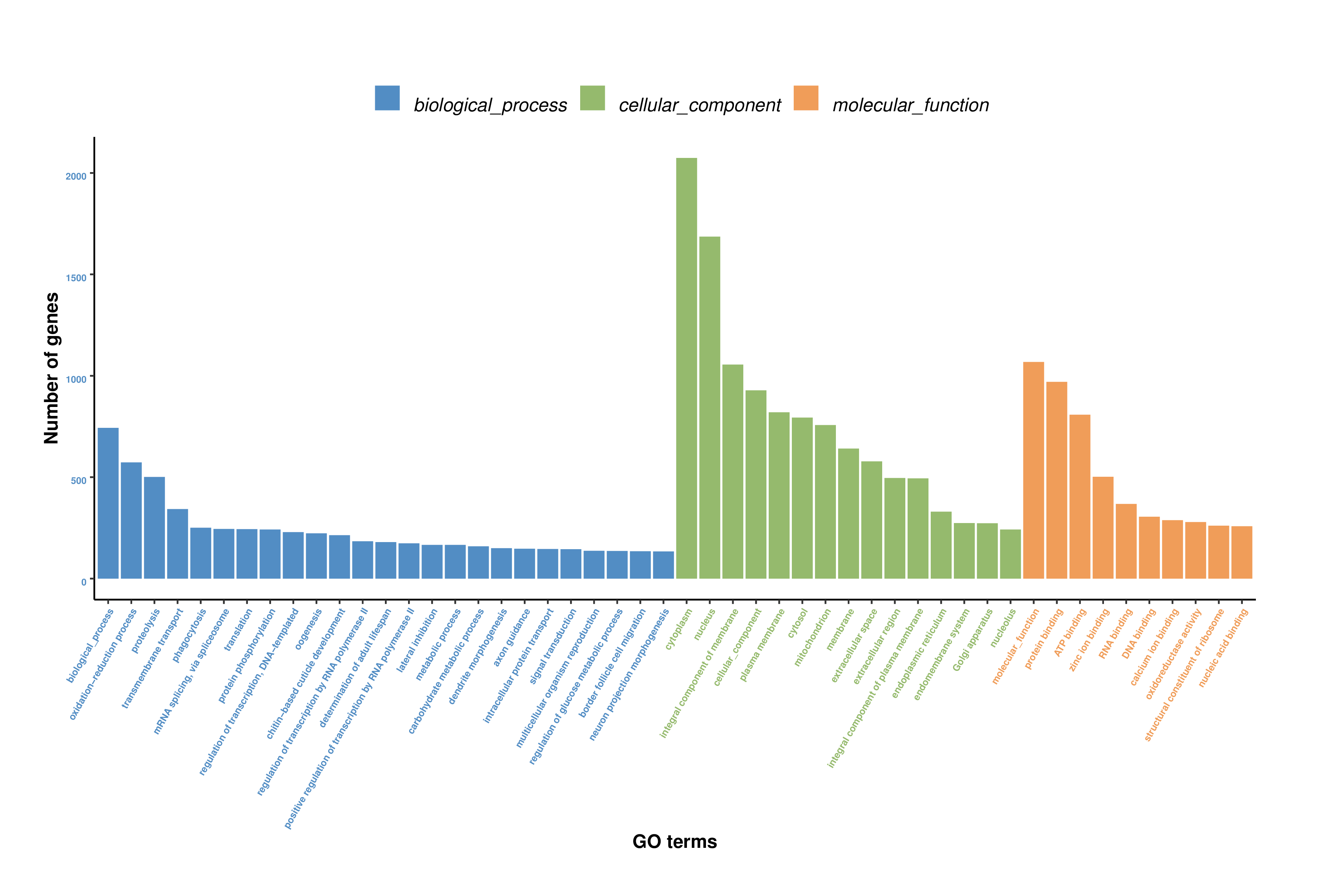

Supplement: Supplementary file 1 [file insects-15-00090-s001.zip › annotation/GO/GO_category.png]

# KEGG Pathway Classification

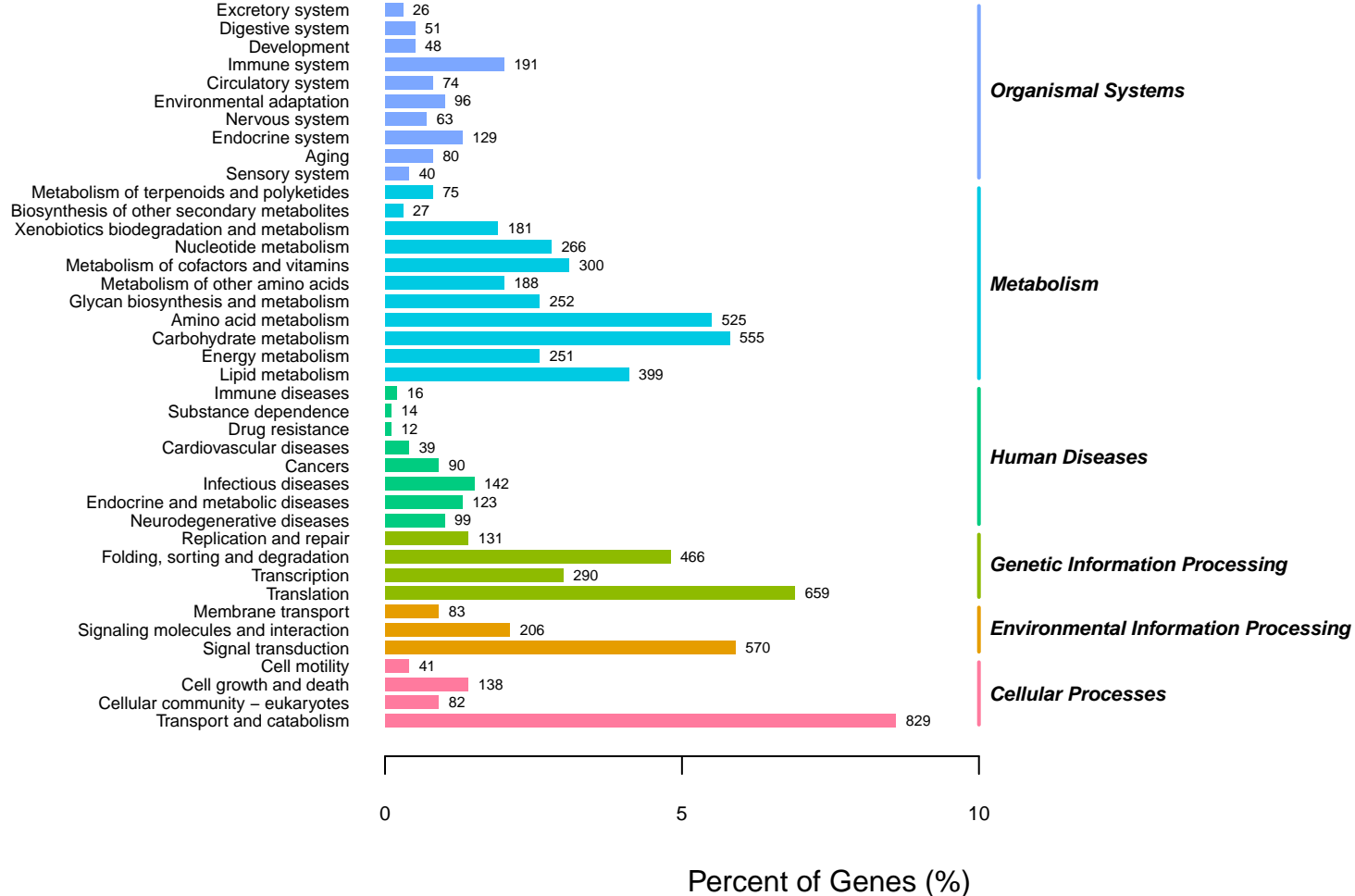

Supplement: Supplementary file 1 [file insects-15-00090-s001.zip › annotation/KEGG/KEGG_category.pdf]

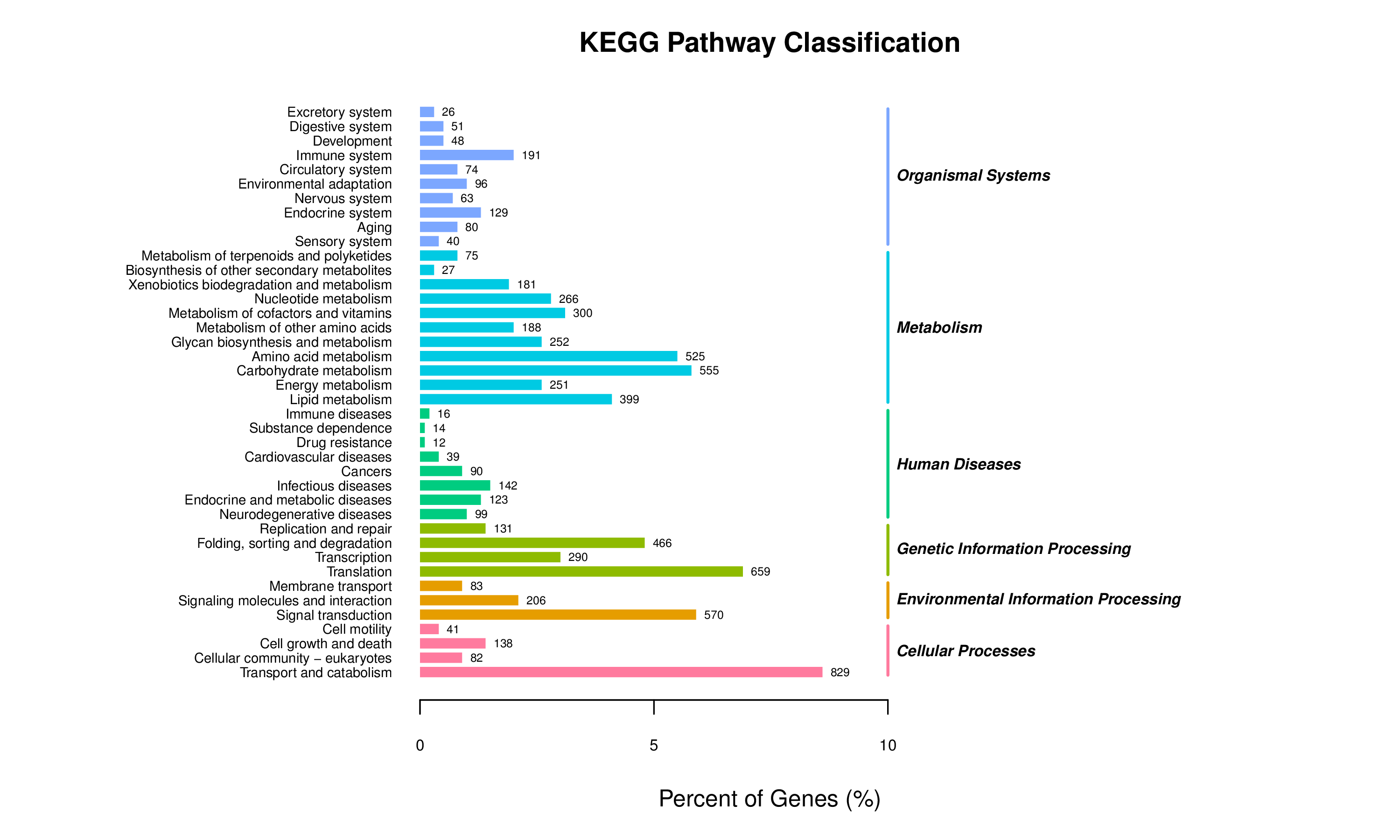

Supplement: Supplementary file 1 [file insects-15-00090-s001.zip › annotation/KEGG/KEGG_category.png]

## Species Distribution

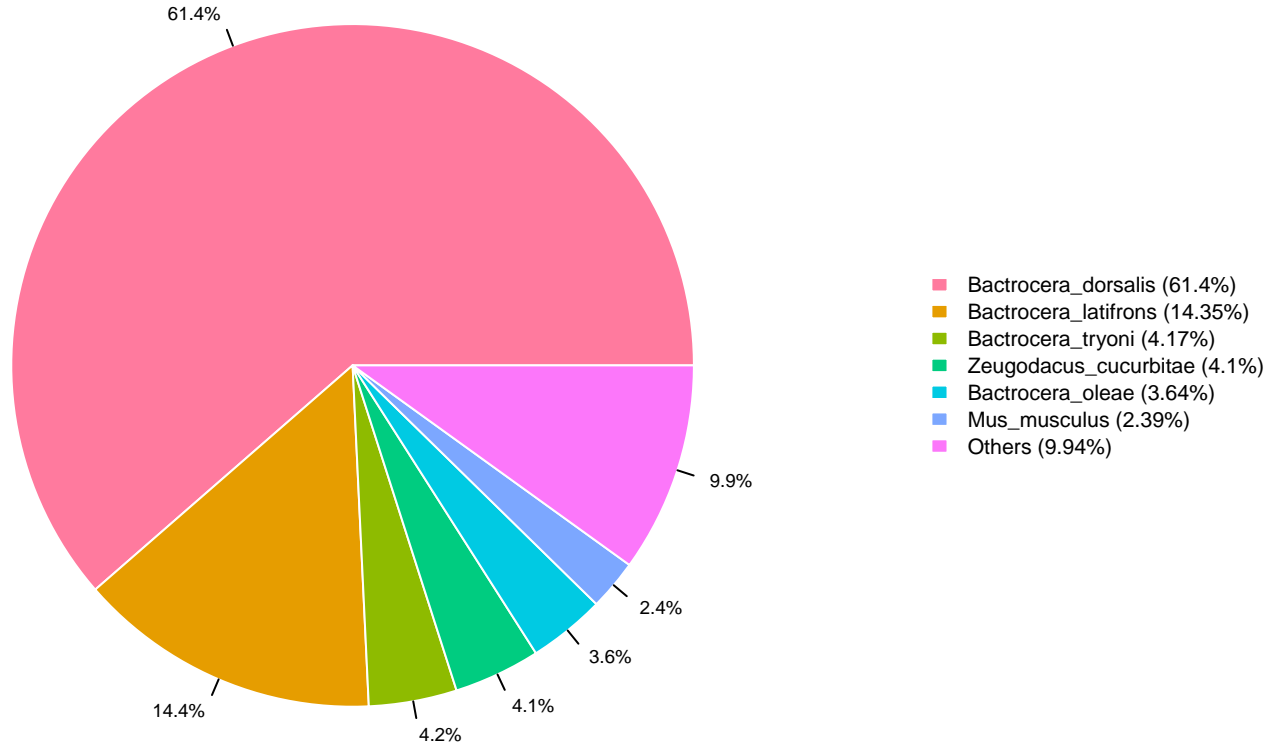

Supplement: Supplementary file 1 [file insects-15-00090-s001.zip › annotation/NR/gene_blast_species.pdf]

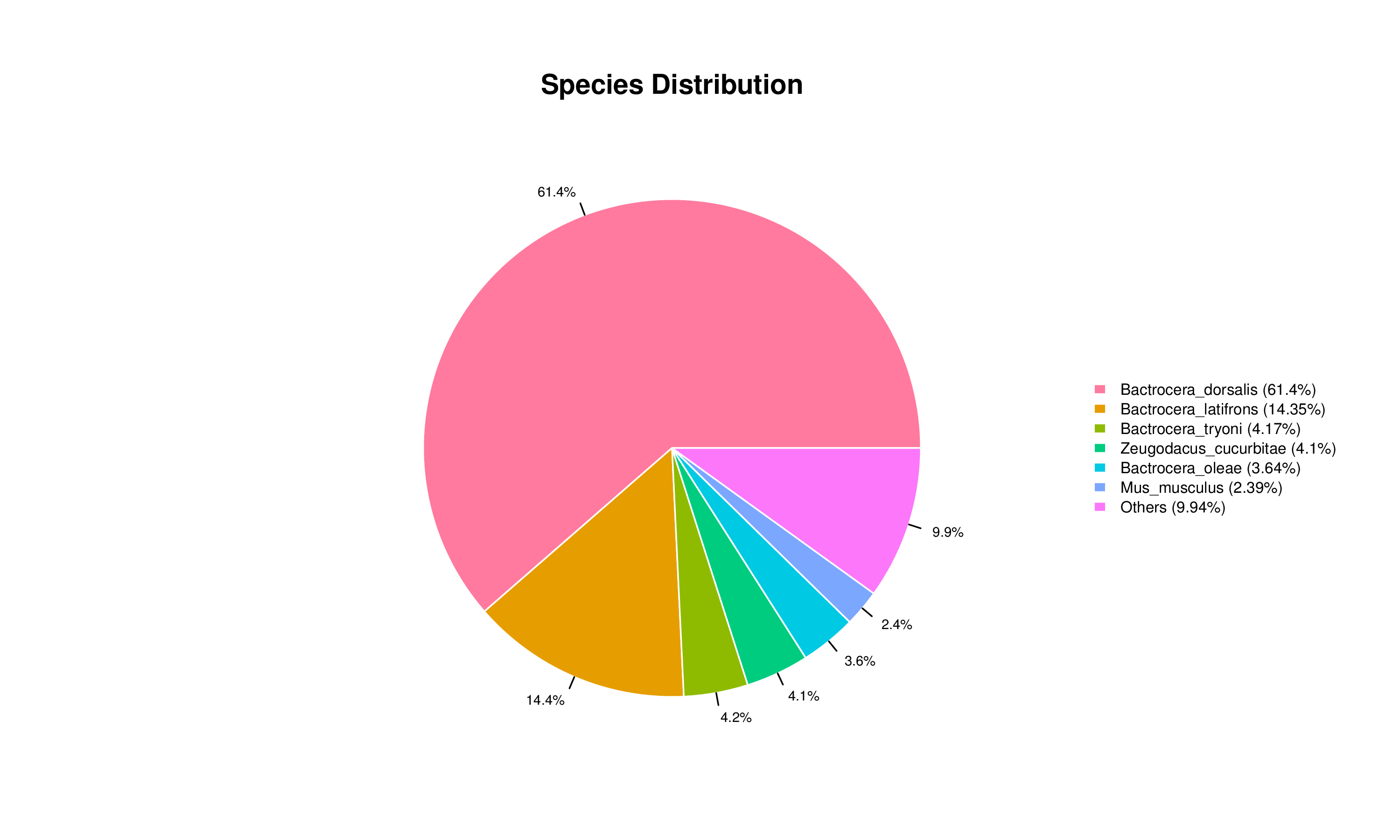

Supplement: Supplementary file 1 [file insects-15-00090-s001.zip › annotation/NR/gene_blast_species.png]

GC Content Distribution

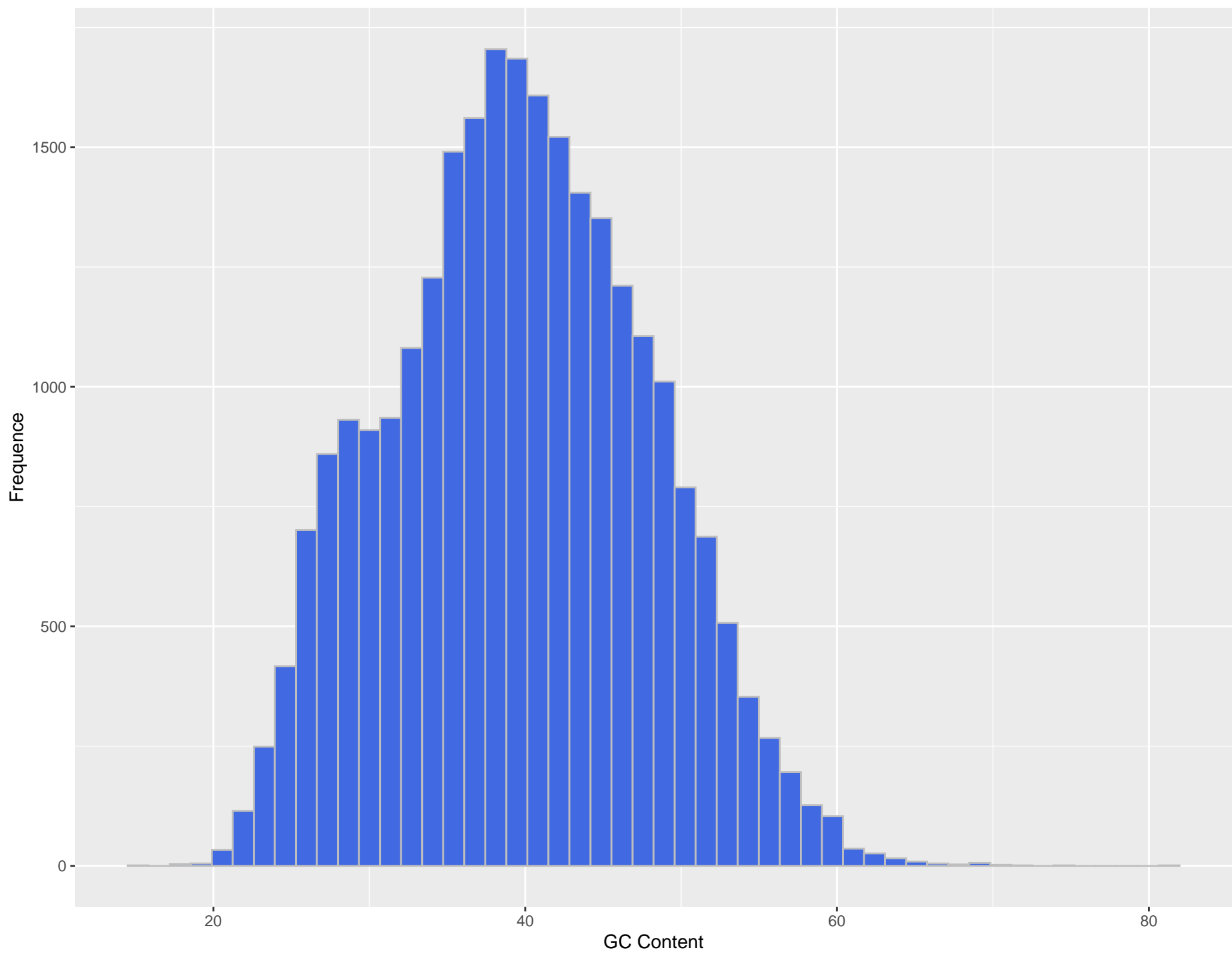

Supplement: Supplementary file 1 [file insects-15-00090-s001.zip › assembly/Trinity.gene.gc.content.pdf]

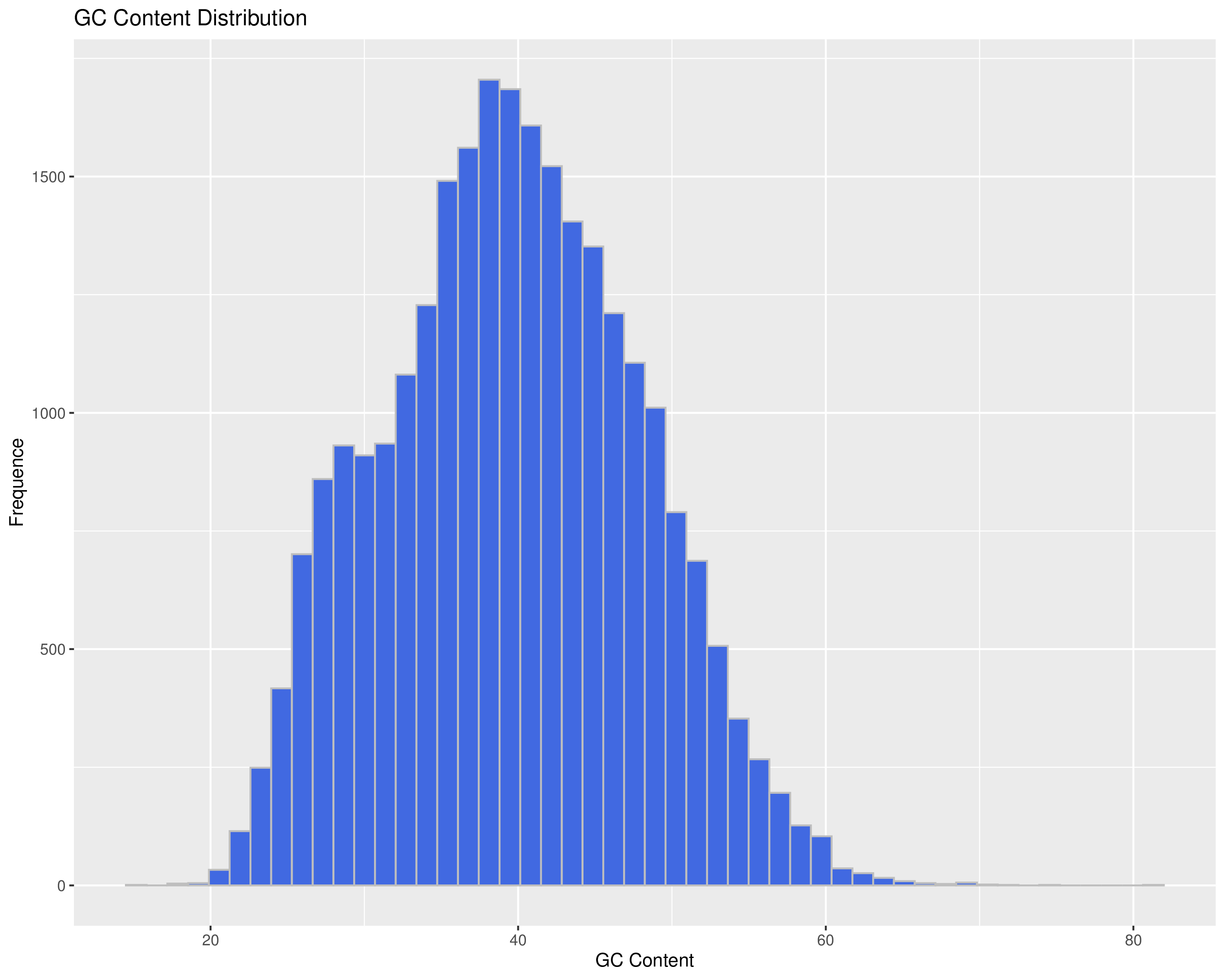

Supplement: Supplementary file 1 [file insects-15-00090-s001.zip › assembly/Trinity.gene.gc.content.png]

# Length Distribution of Genes

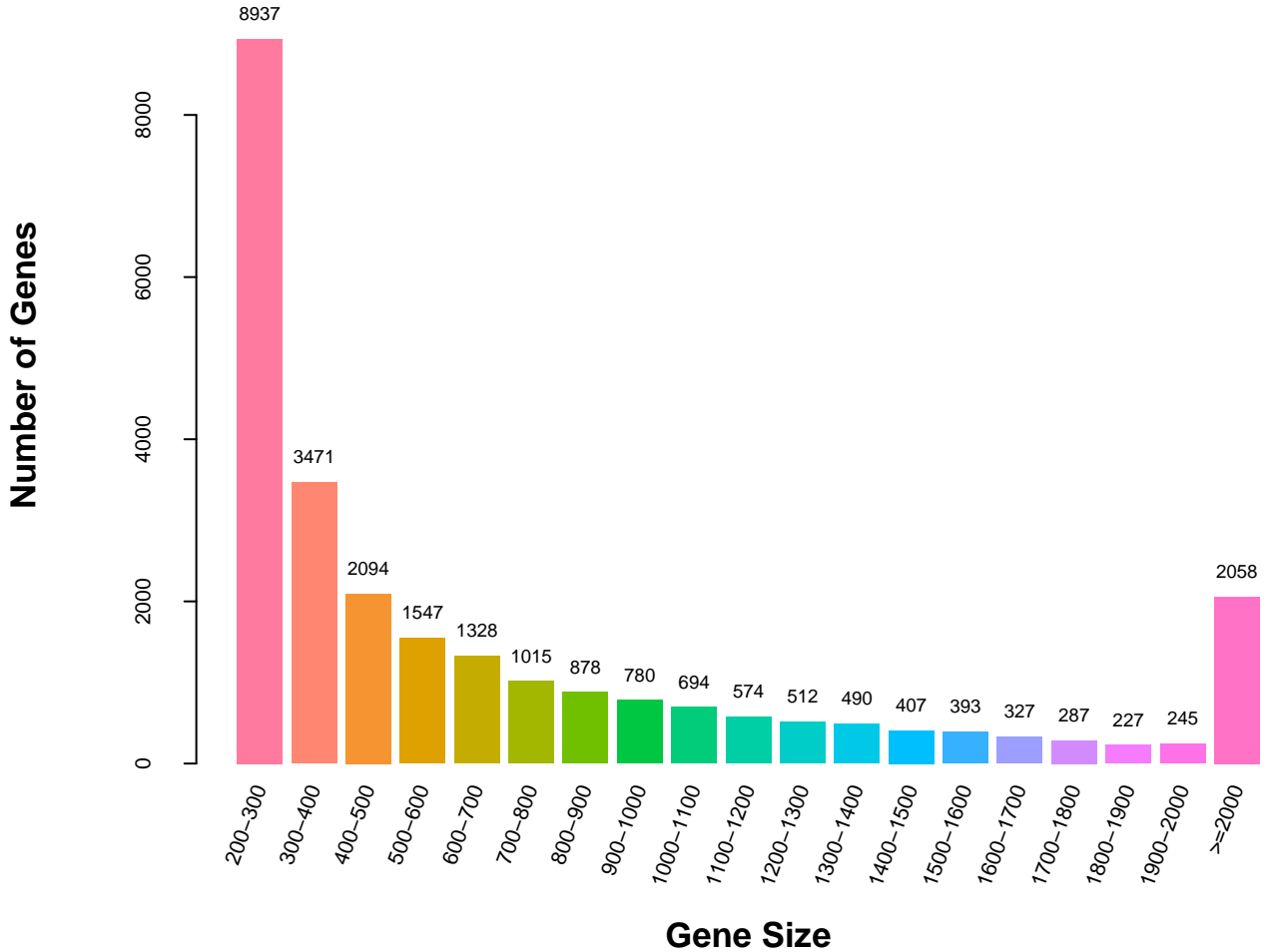

Supplement: Supplementary file 1 [file insects-15-00090-s001.zip › assembly/Trinity.gene.len.dist.pdf]

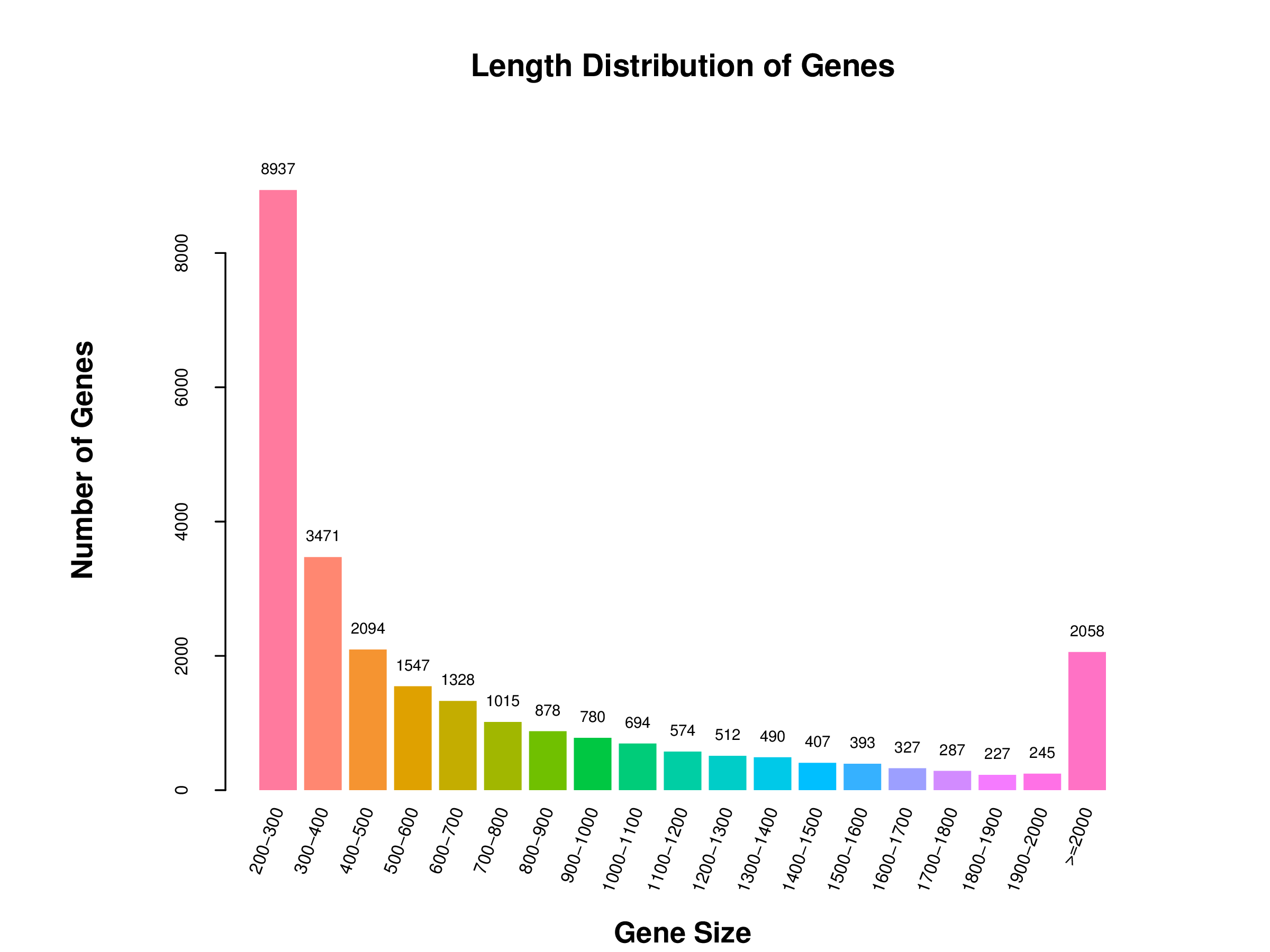

Supplement: Supplementary file 1 [file insects-15-00090-s001.zip › assembly/Trinity.gene.len.dist.png]

GC Content Distribution

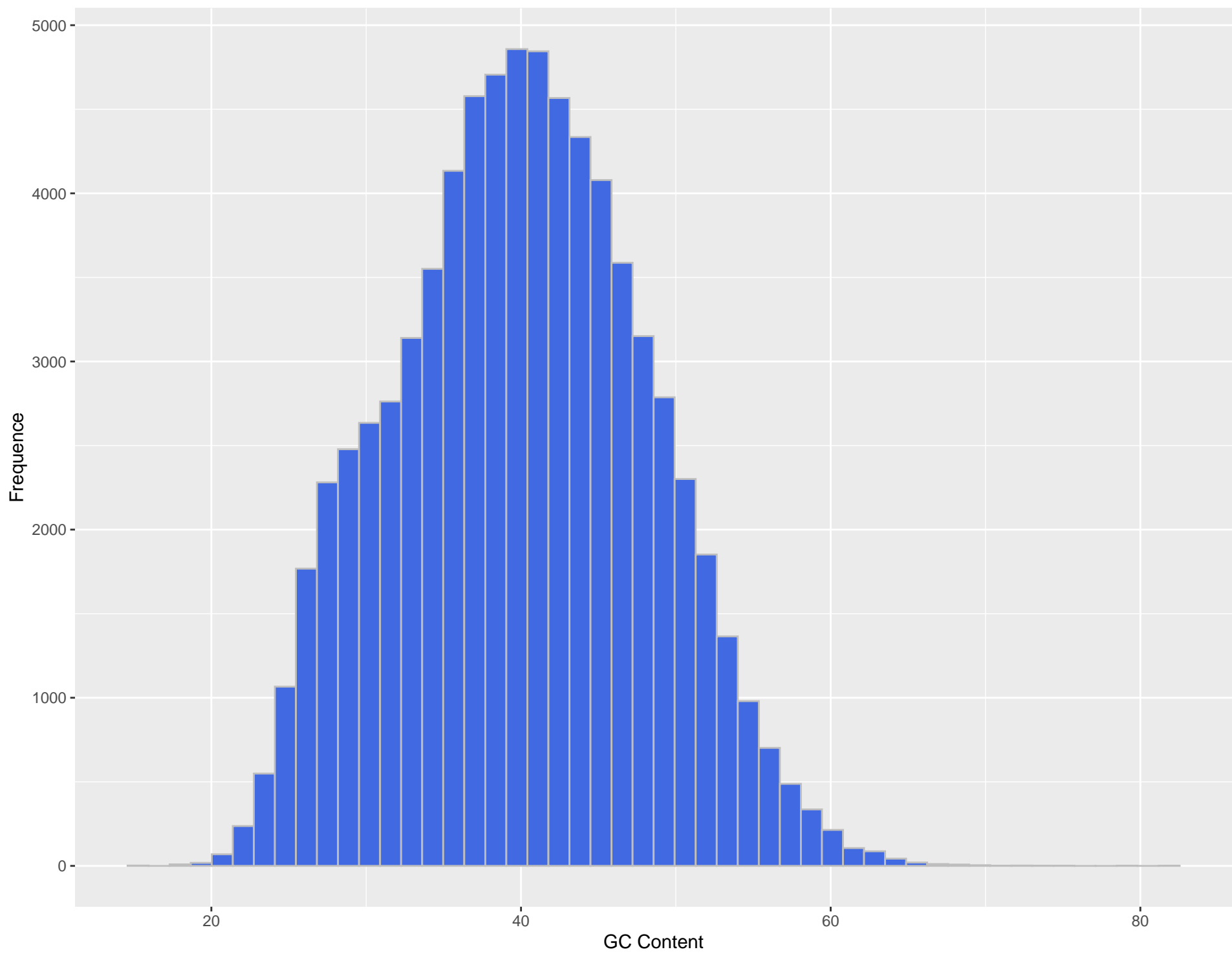

Supplement: Supplementary file 1 [file insects-15-00090-s001.zip › assembly/Trinity.trans.gc.content.pdf]

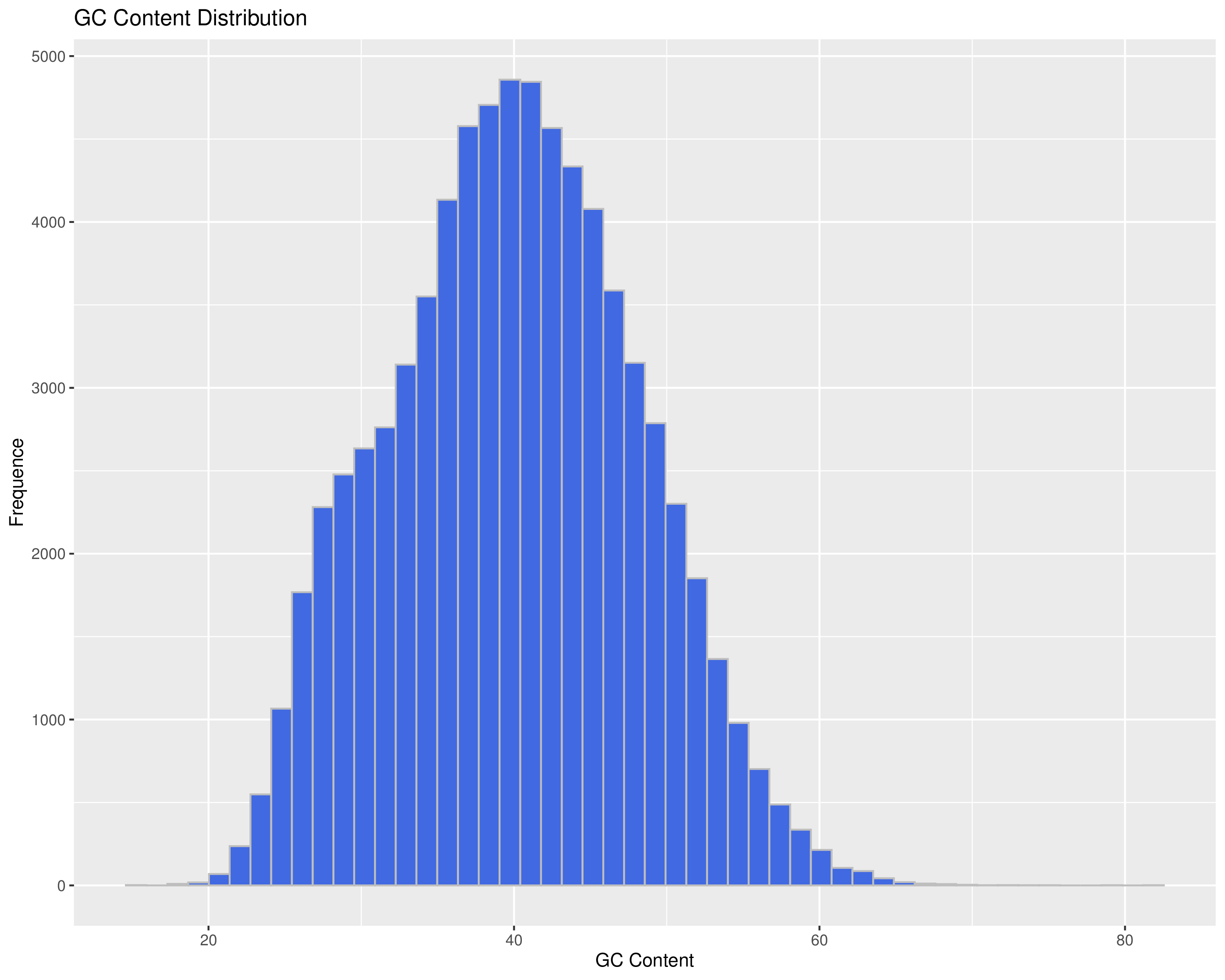

Supplement: Supplementary file 1 [file insects-15-00090-s001.zip › assembly/Trinity.trans.gc.content.png]

# Length Distribution of Transcripts

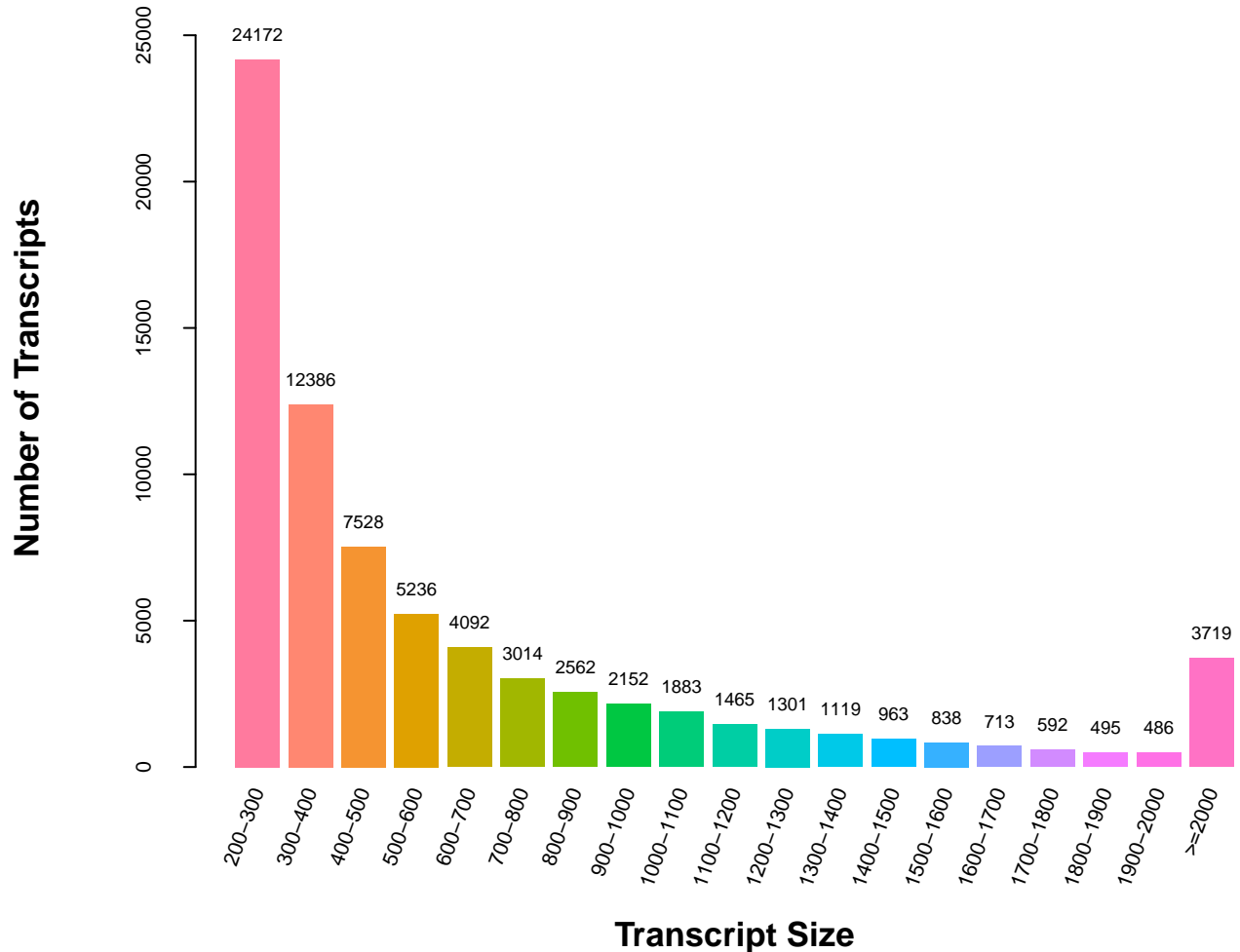

Supplement: Supplementary file 1 [file insects-15-00090-s001.zip › assembly/Trinity.trans.len.dist.pdf]

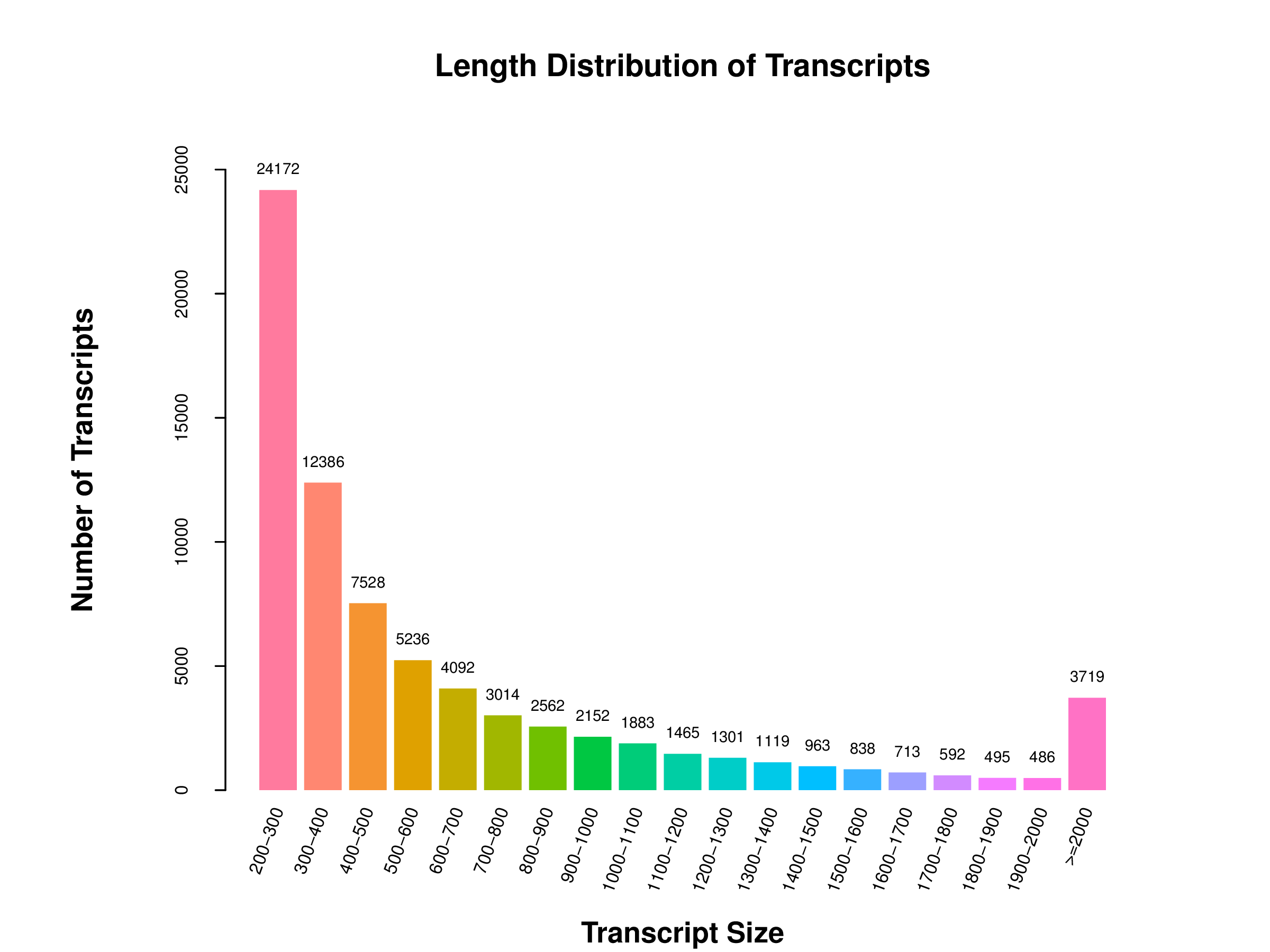

Supplement: Supplementary file 1 [file insects-15-00090-s001.zip › assembly/Trinity.trans.len.dist.png]

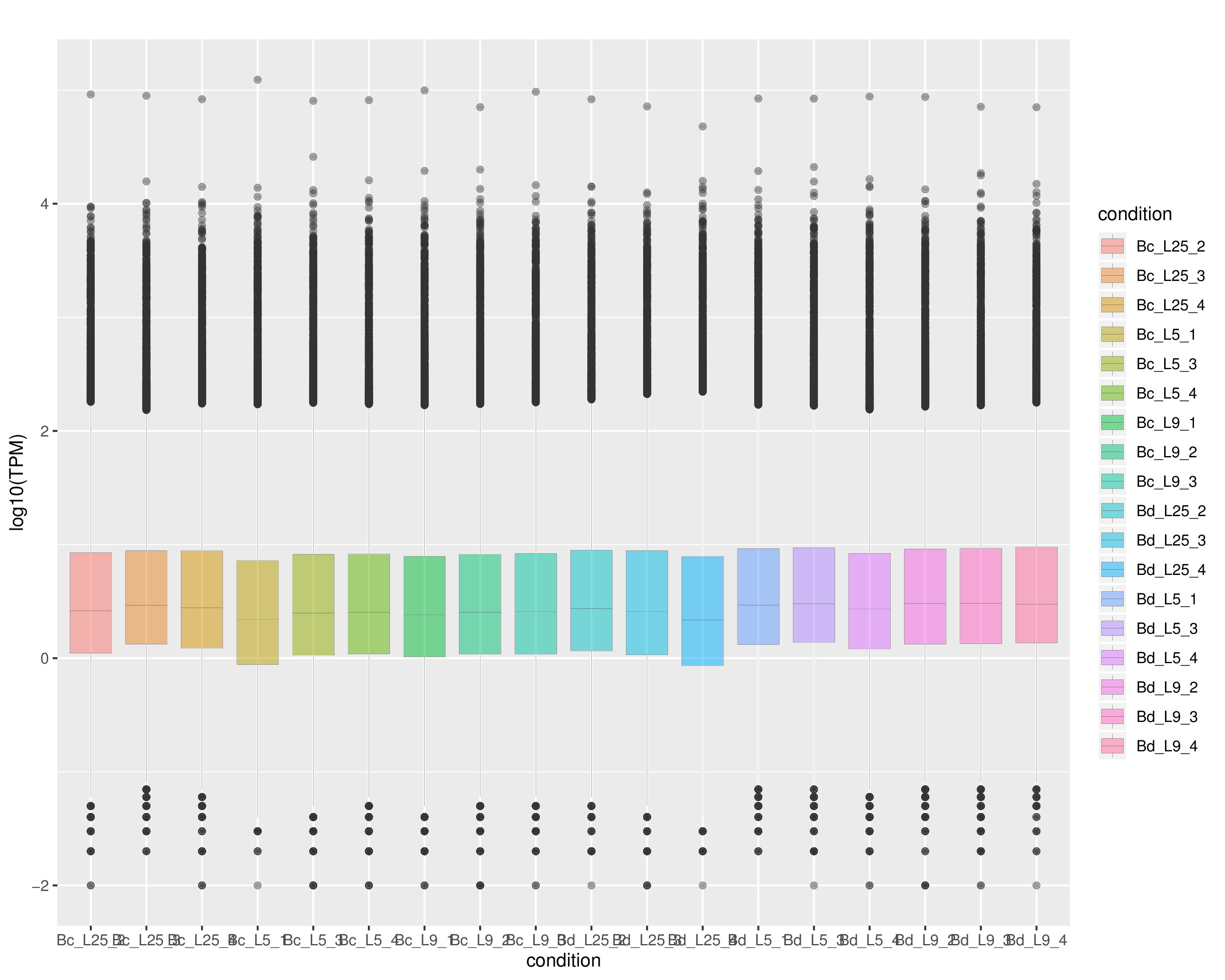

Supplement: Supplementary file 1 [file insects-15-00090-s001.zip › gene_expression/gene_expression_boxplot.png]

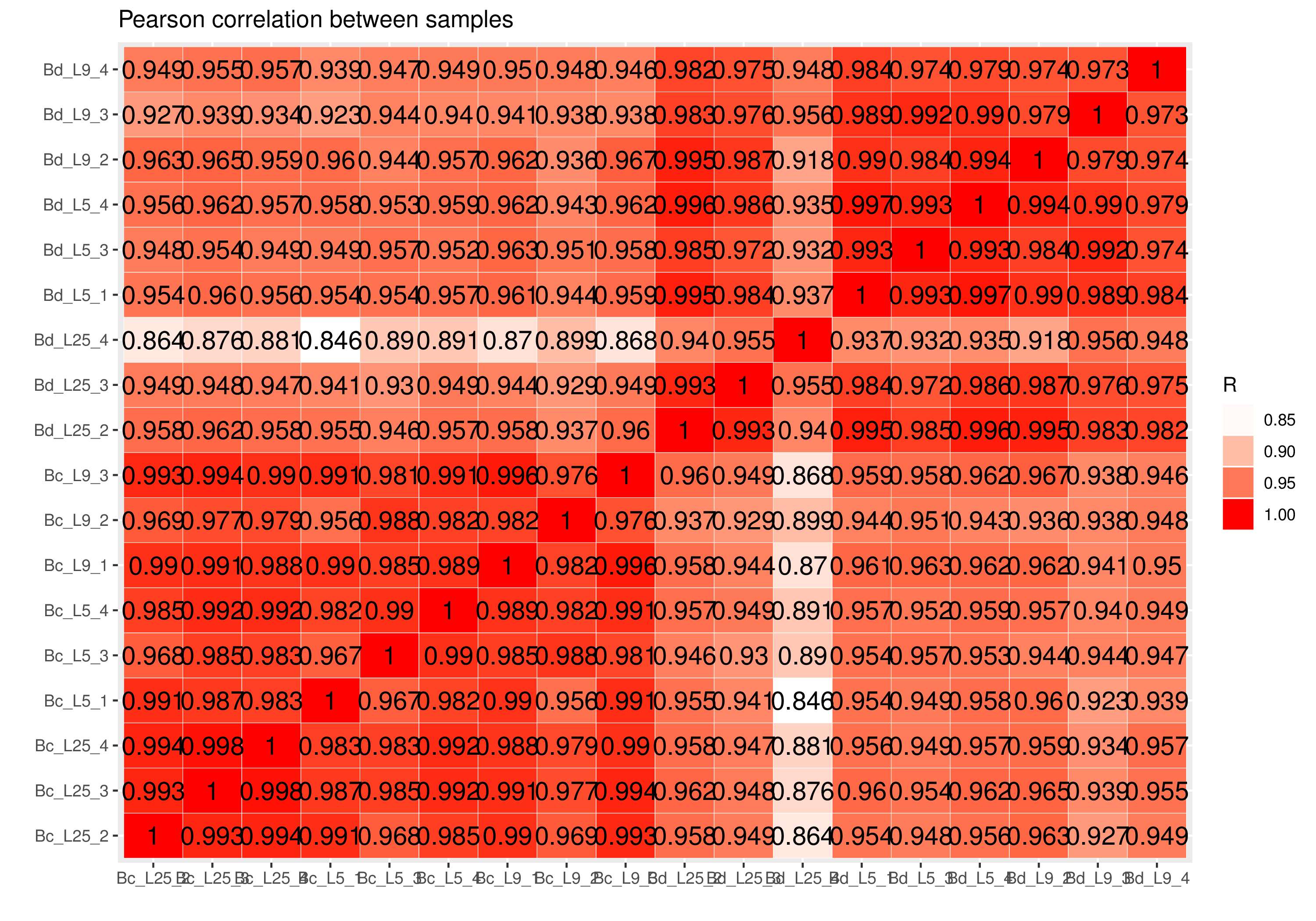

Supplement: Supplementary file 1 [file insects-15-00090-s001.zip › gene_expression/gene_expression_correlation_tile_plot.png]

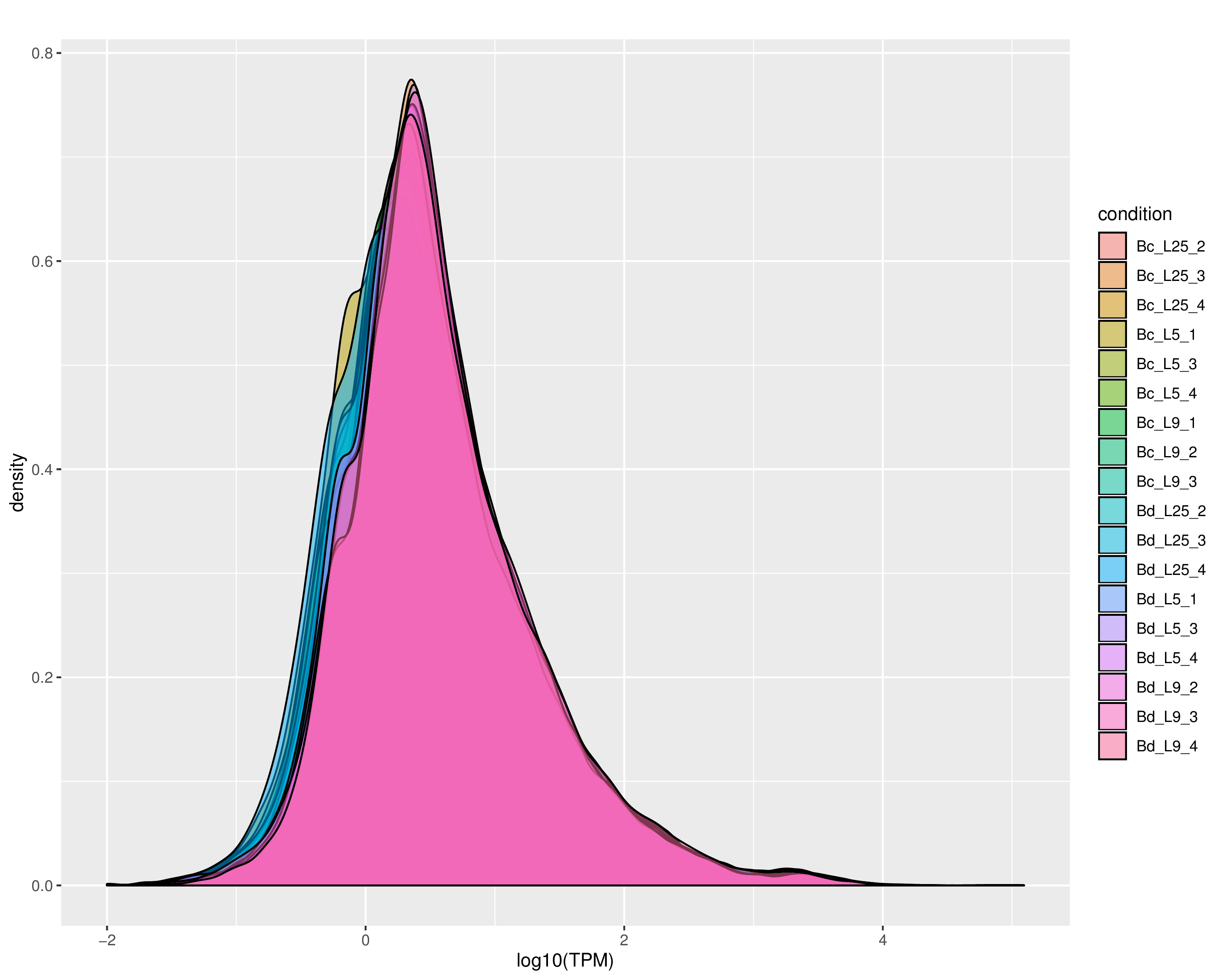

Supplement: Supplementary file 1 [file insects-15-00090-s001.zip › gene_expression/gene_expression_density_plot.png]
